# miR-146a-Enriched Extracellular Vesicles Attenuate Neuroinflammation and Promote Recovery After Ischemic Stroke

**DOI:** 10.5812/ijpr-167245

**Published:** 2026-04-15

**Authors:** Nana Hao, FuHua Gong

**Affiliations:** 1 Department of Neurology, Xi'an Daxing Hospital, Xi'an, China; 2 Department of Neurology, Xi’an People’s Hospital (Xi’an Fourth Hospital), Xi’an, China

**Keywords:** Ischemic Stroke, Extracellular Vesicles, miR-146a, Neuroinflammation, Cytokines, RT-PCR, Bederson Scale

## Abstract

**Background:**

Ischemic stroke triggers a strong neuroinflammatory response involving cytokines, which are fundamentally involved in tissue damage and repair.

**Objectives:**

The present study was designed to investigate the therapeutic potential of miR-146a-enriched extracellular vesicles derived from bone marrow mesenchymal stem cells (BMSC-EVs) in a rat model of middle cerebral artery occlusion (MCAO).

**Methods:**

miR-146a was transfected into BMSCs using Lipofectamine 3000. Extracellular vesicles (EVs) were isolated from transfected BMSCs using ultracentrifugation. Thirty rats were randomly assigned to three groups (n = 10 per group): (1) Control (received FBS), (2) miR-control EVs (miR-NC), and (3) miR-146a-enriched EVs, administered intravenously post-ischemia. Neurological deficits were evaluated with the Bederson score, and cytokine levels, including tumor necrosis factor-α (TNF-α), interferon-γ (IFN-γ), interleukin (IL)-6, IL-10, as well as transforming growth factor β (TGF-β), were measured in splenocyte culture and brain tissues using enzyme-linked immunoassay (ELISA) and reverse transcription-polymerase chain reaction (RT-PCR), respectively. The expression of Interleukin-1 Receptor-Associated Kinase 1 (IRAK1), Tumor Necrosis Factor Receptor-Associated Factor 6 (TRAF6), and nuclear factor kappa B (NF-κB) was also measured using RT-PCR.

**Results:**

Results showed that EVs enriched in miR-146a significantly decreased pro-inflammatory cytokines, TNF-α, IFN-γ, and IL-6, and increased anti-inflammatory markers, IL-10 and TGF-β, when compared with miR-control EVs and FBS-treated groups. miR-146a-EV-treated rats also displayed better Bederson scores, which reflected better neurological recovery. Results also demonstrated that miR-146a-enriched EVs downregulated IRAK1, TRAF6, and NF-κB signaling in ischemic brain tissue.

**Conclusions:**

These results indicate that miR-146a overexpression in BMSC-EVs can significantly inhibit neuroinflammation and promote functional improvement in ischemic stroke models, supporting its targeted therapeutic potential.

## 1. Background

Globally, stroke remains a paramount health challenge, standing as a primary source of lasting disability in the developed world and a leading cause of death (1). Ischemic stroke is the most common type and happens when an artery in the brain is blocked. These blockages are most commonly caused by one of three main mechanisms: A clot that develops in a cerebral vessel (thrombosis), a clot that travels to the brain from somewhere else, such as the heart (embolism), or a significant obstruction of an artery (stenosis) (2).

For patients experiencing an acute ischemic stroke, the current standard of care for dissolving clots is a thrombolytic drug administered intravenously called alteplase. This intervention, called intravenous thrombolysis (IVT), may be used alone or in combination with a mechanical thrombectomy (endovascular treatment, or EVT) in cases in which a major vessel is blocked, usually within a critical six-hour window from symptom onset. However, one major concern that may discourage treating clinicians from IVT is the potential of IVT to cause serious bleeding complications, in particular, symptomatic intracerebral hemorrhage (ICH) (3). These key limitations of the current standard of care highlight the urgent need for new treatment approaches that are able to enlarge the therapeutic time window and increase safety.

However, the injury process continues long after the initial clot is removed. A prolonged neuroinflammatory response then takes hold, driving secondary brain damage that can worsen outcomes long after the stroke itself. This response is characterized by the dysregulated release of pro- and anti-inflammatory cytokines (4). Therefore, finding ways to calm this damaging inflammation presents a major opportunity for improving recovery.

Cell-free therapy, and in particular mesenchymal stem cell-derived extracellular vesicles (EVs), have therefore gained interest as a potential alternative. These small vesicles serve as natural delivery vehicles, delivering healing molecules into cells without the downsides of whole-cell transplantation. Their action depends on the anti-inflammatory effects and tolerance induced by some microRNAs (miRNAs) (5). microRNAs are short non-coding RNA strands, which are capable of directly binding with several mRNAs and are potential regulators of whole signaling pathways rather than single protein targets. Thanks to this fine-tuning mechanism, miRNAs are known to control complex biological processes, including inflammation, apoptosis, and tissue repair (6). But their instability and rapid degradation in vivo prevent miRNAs from serving as therapeutic agents (7). Encapsulation of miRNAs into EVs protects them from enzymatic degradation and facilitates their cellular uptake and bioavailability, making the EV-mediated delivery of miRNAs a potential strategy to modulate post-stroke inflammation and recovery (8, 9).

Studies have shown that miR-146a serves as a master coordinator of inflammation, silencing inflammation by targeting specific molecules such as Interleukin-1 Receptor-Associated Kinase 1 (IRAK1) and Tumor Necrosis Factor Receptor-Associated Factor 6 (TRAF6). This action ultimately suppresses the broader nuclear factor kappa B (NF-κB) pathway, which is responsible for driving the production of damaging pro-inflammatory cytokines (10). Furthermore, studies in animal models showed that when secreted by mesenchymal stem cells (MSCs), this miRNA (miR-146a-5p) not only calms this harmful inflammation but also actively promotes healing and tissue repair (11). In addition, the beneficial effects of miR-146a delivered by MSC-derived extracellular vesicles (MSC-EVs) to reduce inflammation have been positively corroborated in previous studies in mice, suggesting that it may represent a potential therapy to intervene in a wide range of diseases, including retinitis pigmentosa (12), spinal cord injury (13), and acute lung injury (14).

Although these promising findings from other injury models exist, little effort has been made to investigate the possibility of miR-146a-rich EVs being harnessed to shift the detrimental inflammatory landscape in the brain after stroke insult.

## 2. Objectives

Based on this rationale, we postulated that miR-146a-enriched bone marrow MSC-EVs (BMSC-EVs) would exert robust anti-neuroinflammatory effects and improve functional recovery in a rat model of ischemic stroke.

## 3. Methods

### 3.1. Ethics Approval

All experimental procedures were conducted in full compliance with the ARRIVE (Animal Research: Reporting of in vivo Experiments) guidelines and in accordance with the ethical standards outlined in the Guide for the Care and Use of Laboratory Animals (National Academy of Sciences, National Institutes of Health Publication No. 86-23, revised 1985). The Ethics Committee of Daxing Hospital approved this study (ID:20250416).

### 3.2. BMSC Isolation

BMSCs were isolated from bone marrow aspirates of healthy donors after informed consent and approval of the local institutional review board and ethics committee, according to the Declaration of Helsinki. Mononuclear cells were separated using Ficoll density gradient centrifugation and resuspended in MSC basal medium. Approximately 2 × 10⁶ cells/well were seeded and cultured in the MSC basal medium containing 10% human platelet lysate, 1% L-glutamine, and 1% penicillin-streptomycin. Cultures were maintained at 37°C in a humidified 5% CO₂ incubator. Adherent cells showing spindle-shaped, fibroblast-like morphology were cultured, and non-adherent cells were removed by changing the medium after 24 h.

At 2 - 3-day intervals, culture medium was replaced, and when the cells reached 80 - 90% confluence, they were subcultured by trypsinization as per standard protocol. BMSCs were passaged 1:3 and used for experimentation between passages 3-5 to allow for phenotype stability and to minimize variability due to senescence (15).

### 3.3. Characterization of Isolated BMSCs

In order to identify the BMSCs, the surface markers were detected using flow cytometry. The cells were positive for CD73 and CD105 but negative for CD45. Their multipotent differentiation potential was also examined. Adipogenic differentiation, induced for two weeks with 10 ng/ml insulin and 1 × 10 - 8M dexamethasone, was confirmed by Oil Red O staining. Osteogenic differentiation (a three-week treatment with 50 μg/ml L-ascorbic acid-2 phosphate, 10mM glycerol 2-phosphate disodium salt, and 1 × 10 - 8M dexamethasone) was confirmed by Alizarin Red staining.

### 3.4. Transfection of BMSCs with miR-146a

For transfection, BMSCs were transfected with Lipofectamine 3000 reagent (Invitrogen, USA) according to the manufacturer's protocol. Briefly, BMSCs were expanded to 80% confluence. Subsequently, the medium was replaced with Opti-MEM, and Lipofectamine 3000-miRNA complexes were added. These complexes carried either a miR-146a mimic or a non-targeting miRNA mimic (miR-NC). The transfection mixture was added to the cells and incubated for 72 h to maximize cellular uptake and expression of the miRNA.

To obtain EVs with customized miRNA content, BMSCs were set up in two different experimental conditions before EV isolation. For the miR-146a-enriched EVs, BMSCs were transfected with the miR-146a mimic as above. BMSCs were also used to generate miR-control EVs by transfecting them with a non-targeting miR-NC using the same transfection reagent, concentration, and incubation process to normalize the potential effect of the transfection procedure itself.

### 3.5. Isolation and Characterization of Extracellular Vesicles from BMSC-Enriched miR-146a

BMSC-EVs were obtained from the conditioned medium of BMSCs through differential ultracentrifugation, a commonly used technique for EV isolation. When BMSCs were 80 - 90% confluent, the medium was replaced with medium containing EV-depleted fetal bovine serum (FBS) to prevent contamination with bovine-derived vesicles. The cells were then cultured in normal culture medium (37°C, 5% CO_2_) for 48 h for EV secretion.

Extracellular vesicles -depleted FBS was processed by centrifugation to pellet the preexisting vesicles. Briefly, FBS was centrifuged at 500 × g for 20 min to eliminate cellular debris and at 18,000 × g for 30 min. The supernatant was subjected to an additional spin at 120,000 × g for 7 h. To maintain the quality of the serum, all the centrifugations were done at 4°C.

The culture medium was harvested and centrifuged. Removal of residual cells and debris was performed by centrifugation at 500 × g for 20 min and at 18,000 × g for 30 min at 4°C. The supernatant was subsequently filtered through a 0.22 μm sterile filter to remove any remaining particles and then spun at 120,000 × g for 90 min to pellet the EVs. The final EV pellet was resuspended in sterile PBS and processed for downstream analysis.

To characterize the isolated EVs, we confirmed the presence of a prototypical EV membrane protein by measuring CD9 tetraspanin marker expression. This was performed with an EV isolation kit based on flow cytometry (EV Isolation Kit CD9, Miltenyi Biotec, Germany). We also performed a bicinchoninic acid (BCA) assay (Solarbio, Beijing, China) to determine the total protein content of the EV preparation for further accurate EV dose normalization. Additionally, the size distribution and mean diameter of EVs were also analyzed by the HORIBA SZ-100 system. The measurements were carried out at the 173° scattering angle using the 30% neutral density filter. The sample was resuspended in PBS and kept at 25.2°C before analysis. Data analysis was performed using HORIBA software (version 2.20).

### 3.6. Rat Model of Middle Cerebral Artery Occlusion

A total of thirty healthy male Sprague-Dawley rats (6 - 8 weeks old, 200 ± 20 g) were kept under specific-pathogen-free conditions. Animals were kept at a room temperature of 24°C with a 12 h light/dark cycle and had free access to standard rodent chow and water. In order to encourage natural behavior and well-being, rats were housed in groups (3 to 4 per cage) on woodchip bedding with nesting material and cardboard tunnels for environmental enrichment. According to the principles of ARRIVE 2.0 guidelines, the knowledgeable staff of the animal facility thoroughly carried out daily health examinations, which were documented for grooming, movement, and feeding, respectively.

Rats were anesthetized intraperitoneally with a ketamine/xylazine mix (80 and 10 mg/kg, respectively) to induce focal cerebral ischemia. Following verification of the depth of anesthesia, a midline neck incision was performed, and the right common carotid artery (CCA), external carotid artery (ECA), and internal carotid artery (ICA) were exposed surgically.

A silicone-coated 4 - 0 nylon monofilament suture with a heat-rounded tip was inserted into the ECA and advanced via the ICA into the lumen of the ICA until it blocked the origin of the MCA. The filament remained in place to maintain occlusion for a period of 120 minutes. Following this ischemic interval, it was gently withdrawn to allow reperfusion. All animals were subsequently monitored closely for a 24-hour recovery period (16). The regional cerebral blood flow was observed to verify the occurrence of ischemia by middle cerebral artery occlusion (MCAO), using laser Doppler flowmetry (PeriFlux System 5000, Sweden).

### 3.7. Treatment Protocol

Before MCAO surgery, rats were randomized to three treatment groups (n = 10/group) using a simple random number generator. The experimenters assessing outcome (neurological scoring, collection of tissue, cytokine assays, and qRT-PCR) were blinded to group assignment. Dosing of EVs was normalized on the basis of total protein concentration as measured with BCA. Each rat received 100 µg total EV protein in 200 µL PBS (or an equivalent volume of FBS as vehicle for controls). The dosing was chosen based on previously published preclinical MSC-EV studies, as well as local pilot work, in order to maximize systemic exposure while avoiding volume overload (17, 18). Extracellular vesicles dosing for both miR-control and miR-146a groups was identical to allow comparison of miRNA content administration. A single intravenous dose of the assigned treatment was administered through the lateral tail vein immediately after withdrawing the occluding filament (i.e., immediately after reperfusion).

### 3.8. Regional Cerebral Blood Flow

Cerebral blood flow was monitored during the procedure with laser Doppler flowmetry. It offers continuous real-time estimation of microvascular perfusion based on monitoring the Doppler shift in laser light reflected from moving red blood cells. The head of an anesthetized animal was fixed, and a specially designed probe was mounted on the skull over the core territory of the MCA, and capillary blood flow was measured noninvasively and intravenously.

### 3.9. Neurological Deficit Scores

Twenty-four hours following MCAO induction, Neurological Deficit Scores (NDS)were evaluated for all rats using the established Bederson scale (19). The scoring system was applied as follows: A score of 0 represented no observable neurological deficits. A score of 1 was assigned for failure to fully extend the contralateral (left) forepaw. A score of 2 was given when the animal demonstrated reduced resistance to lateral push or showed circling behavior on a flat surface, but maintained a normal posture at rest. A score of 3 indicated spontaneous circling or rolling to the left (ipsilateral side). Finally, a score of 4 was reserved for a severe deficit, characterized by a lack of spontaneous movement or a state of unconsciousness.

### 3.10. Splenocyte Preparation

The spleen was processed into a single-cell suspension by mechanically dissociating the tissue through a 70 μm cell strainer into RPMI-1640 medium (Shellmax, China). To remove red blood cells (RBCs), the resulting suspension was treated with ammonium chloride buffer for 10 minutes on ice. Following lysis of the RBCs, the splenocytes were pelleted by centrifugation at 300 × g at 4°C for 5 min and resuspended in fresh RPMI-1640 to improve purity. Finally, the separated splenocytes were resuspended in complete RPMI-1640 medium containing 10% fetal bovine serum (FBS, Shellmax, China) and 1% penicillin-streptomycin (100 μg/ml, Gibco, UK) to maintain cell viability for the subsequent study.

### 3.11. Cytokine Assay

To evaluate cytokine profiles, isolated splenocytes were plated in 24-well plates (2 million cells per well) followed by 48 h of stimulation with 10 μg/ml phytohemagglutinin (PHA; Gibco). PHA stimulation was applied to activate T lymphocytes and assess cytokine production capacity under standardized conditions. After the incubation, the supernatants from the cell culture were carefully collected. We next measured the levels of an array of important cytokines, including the pro-inflammatory factors tumour necrosis factor-α (TNF-α), interferon-γ (IFN-γ), and interleukin (IL)-6, and the anti-inflammatory cytokines transforming growth factor β (TGF-β) and IL-10, using specific ELISA kits (R&D system, USA). All experiments were carried out with high precision according to the manufacturer's instructions. All samples were analyzed in triplicate to guarantee statistical reliability and precision.

### 3.12. Gene Expression Analysis by qPCR

The mRNA levels of pro-inflammatory cytokines (TNF-α, IFN-γ, and IL-6), anti-inflammatory cytokines (TGF-β and IL-10), and the IRAK1, TRAF6, and NF-κB signaling pathways were assessed by qRT-PCR. Total RNA extracted from the brain homogenates was first isolated using TRIzol reagent. RNA was extracted, and spectrophotometry confirmed the amount and purity of the RNA samples.

For the synthesis of complementary DNA (cDNA), total RNA from each sample (1 µg) was reverse transcribed using a commercially available reverse transcription kit (BioFACT, Korea) following the manufacturer's instructions. The cDNA in each sample was tested using qPCR reactions run with SYBR Green master mix (BioFACT, Korea) and gene-specific primers for the expression of TNF-α, IFN-γ, IL-6, TGF-β, IL-10, IRAK1, TRAF6, NF-κB, and the reference gene GAPDH.

Amplification was carried out using an initial denaturation of 10 min at 95°C, followed by 40 cycles of 15 s at 95°C, and 60 s at 60°C for combined annealing/extension. The internal control GAPDH was used to normalize the Ct value of expression data, and the relative expression levels of genes were determined by the comparative 2^-ΔΔCt^ method.

### 3.13. Statistical Analysis

Statistical methods were applied using IBM SPSS Statistics 26 (IBM Corp., Armonk, NY), and figures were prepared with GraphPad Prism 8 (GraphPad software, USA). All data are expressed as the mean ± standard deviation (SD). The normality of data distribution was assessed using the Shapiro-Wilk test before inferential analysis. For multiple group analyses, parametric (normal distribution) data (such as ELISA-derived cytokine concentrations and cerebral blood flow data) were subjected to one-way ANOVA with Tukey’s post-hoc test for multiple comparisons. Non-parametric RT-PCR data were analyzed by the Kruskal-Wallis test with Dunn's post-hoc comparison with Bonferroni adjustment. Neurological deficit scores (Bederson scale) were analyzed by the Kruskal-Wallis test with post hoc analysis. Longitudinal cerebral blood flow measurements obtained at multiple time points during ischemia and reperfusion were analyzed using two-way repeated-measures ANOVA, followed by Bonferroni’s post hoc test to assess group and time interactions. All statistical tests were two-tailed, and a P-value < 0.05 was considered statistically significant.

## 4. Result

### 4.1. BMSC Characterization

**Figure 1.**
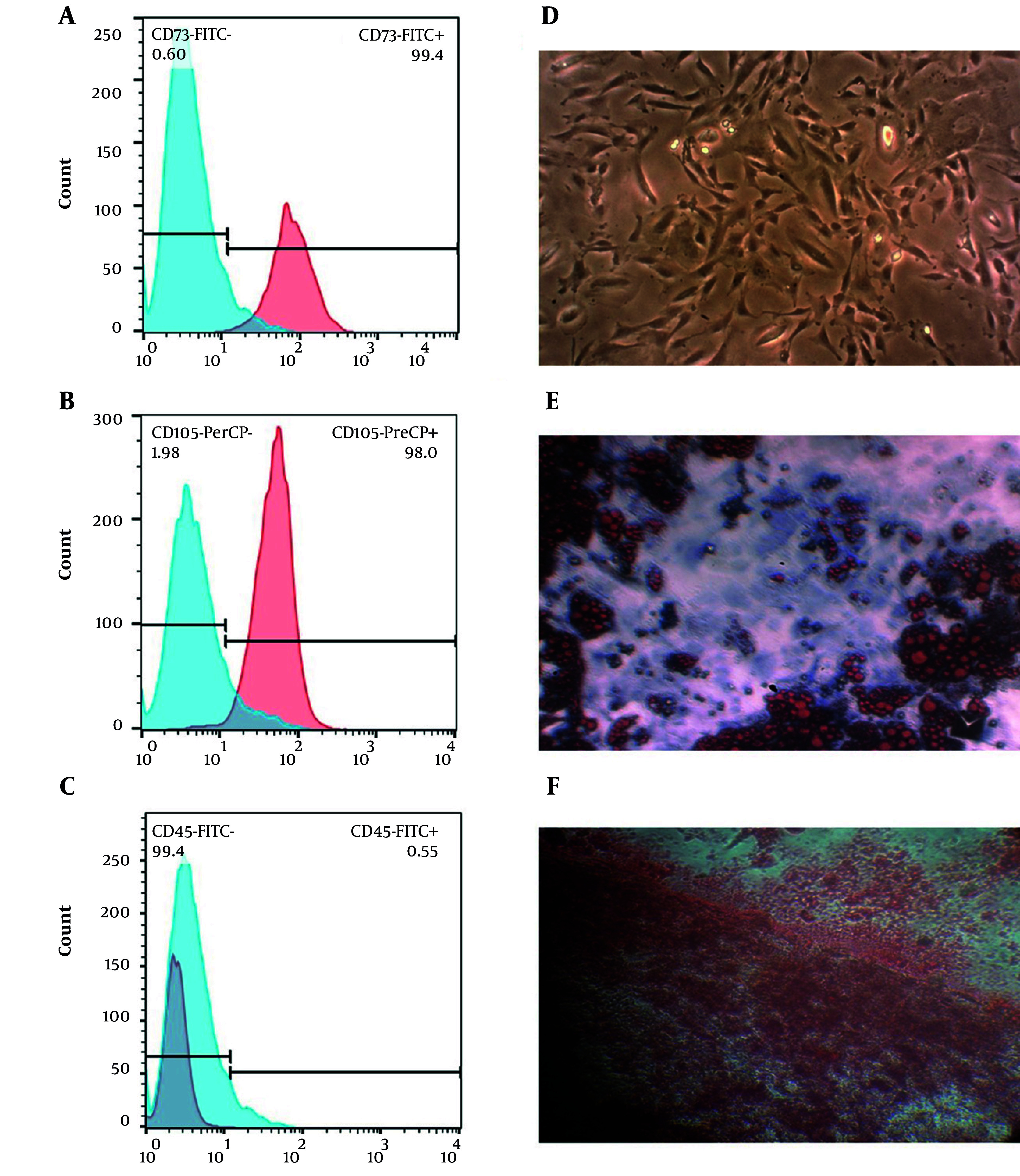
Characterization of BMSCs. Flow cytometric analysis of cultured BMSCs demonstrates high-level expression of the MSC surface markers CD73 (A) and CD105 (B). BMSCs were negative for the hematopoietic antigen CD45 (C). A representative brightfield image of cultured BMSCs with typical spindle-shaped, fibroblast-like morphology (D). Functional evaluation of multipotential differentiation capacity. Lipid droplets after adipogenic differentiation can be observed by Oil Red O staining (E). Formation of calcium deposits after osteogenic differentiation is detected with Alizarin Red staining (F). Abbreviation: BMSC, bone marrow-derived mesenchymal stem cell.

The identity of the BMSCs as mesenchymal stem cells (MSCs) was confirmed by flow cytometry according to their expression of surface antigens: they were CD73- and CD105-positive, but CD45-negative (Figure 1A-C). BMSCs also exhibited a spindle-shaped, fibroblast-like morphology with typical adherent growth. After a 21-day induction, adipogenic differentiation was confirmed by Oil Red O-stained lipid droplets, whereas osteogenic differentiation was verified by Alizarin Red-stained calcium deposits (Figure 1D-F). 

### 4.2. BMSC-EV Characterization

**Figure 2.**
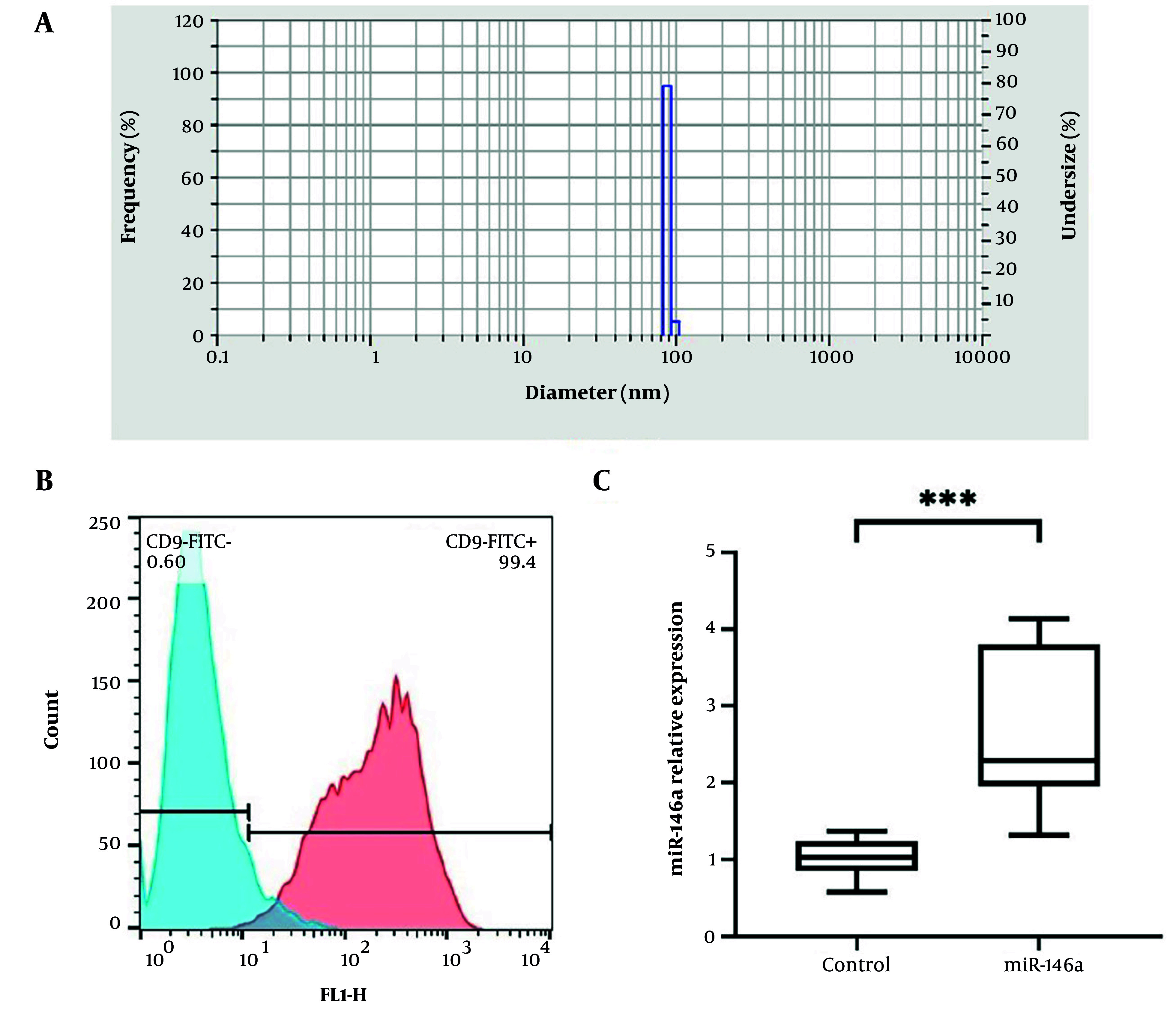
Characterization of EVs derived from BMSCs. The average size of BMSC-EVs, determined by DLS using a HORIBA SZ-100 instrument, was 89.6 nm (A). Flow cytometry analysis confirmed the presence of the EV surface marker CD9 (B). Furthermore, qRT-PCR analysis showed that EVs harvested from miR-146a-transfected BMSCs contained significantly higher levels of miR-146a than control EVs (C). Data are presented as mean ± SD. *** P < 0.001.

To obtain separate EV populations, BMSCs were transfected with miR-146a mimics or negative controls. Extracellular vesicles were then obtained from culture supernatants of these cells following a 48-h incubation. BMSC-EVs were analyzed to ensure their size and identity. Their average size was 89.6 nm as determined by DLS using a HORIBA SZ-100 instrument (Figure 2A). Flow cytometry analysis of these fractions demonstrated that the exosomal surface marker CD9 was enriched, confirming that the isolation was successful and that these preparations were exosomes (Figure 2B). 

### 4.3. Packaging of miR-146a in Extracellular Vesicles

Quantitative RT-PCR results showed that miR-146a was successfully enriched in EVs from transfected BMSCs. EVs isolated from miR-146a mimic-transfected cells exhibited significantly increased levels of miR-146a compared with EVs from control-transfected cells (P = 0.0001), indicating successful miRNA packaging (Figure 2C). 

### 4.4. Effects of miR-146a-Enriched EVs on NDS in MCAO

**Figure 3.**
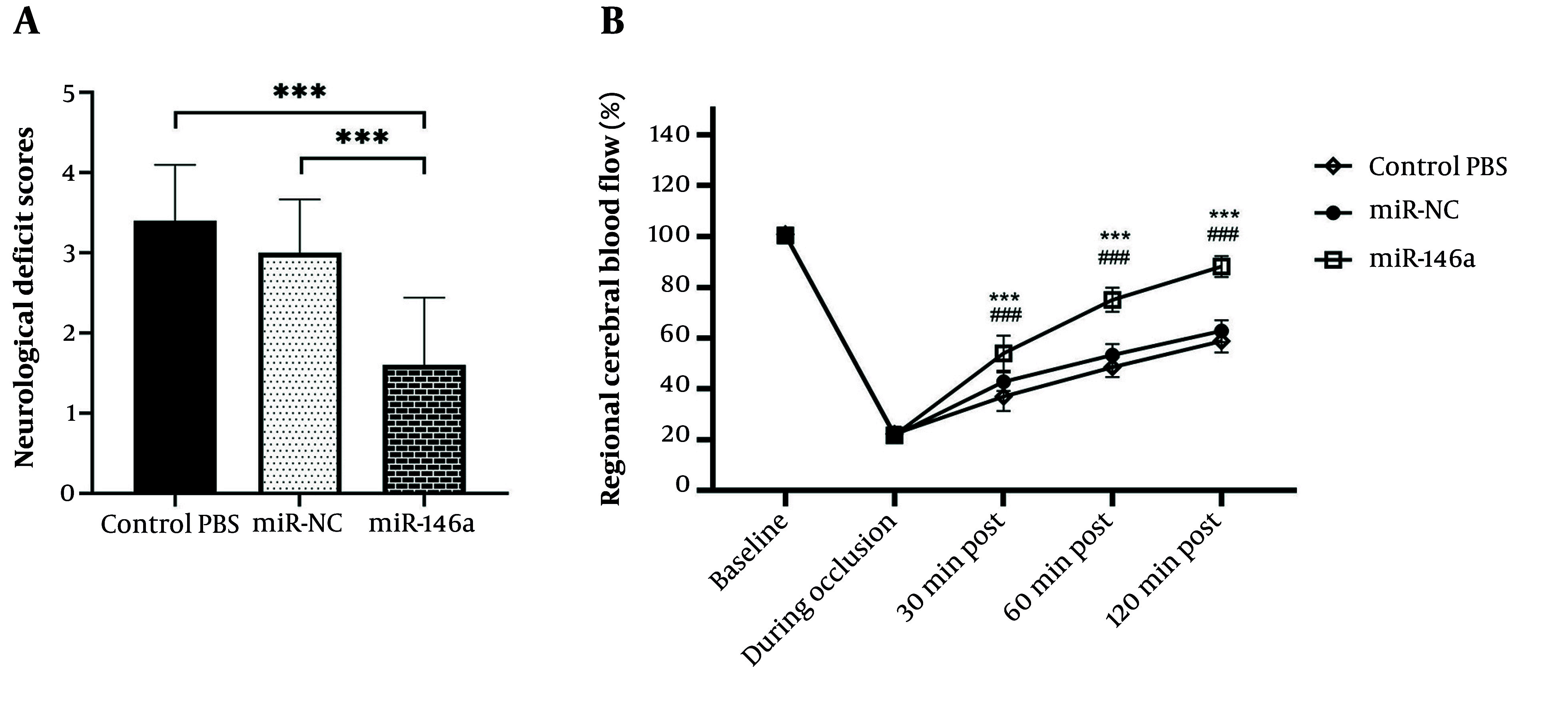
Neurological impairment and CBF recovery by treatment with miR-146a. (A) NDS evaluated by the Bederson Scale 24 hours after reperfusion. miR-146a-treated rats experienced significantly better neurological benefit than the control and miR-NC groups. Data are shown as mean ± SD (n = 10 per group). *** P < 0.001. (B) The time course of regional CBF changes was monitored during ischemia and reperfusion using laser Doppler flowmetry. miR-146a-treated rats had significantly faster and more complete recovery of CBF toward baseline compared with both controls. Data are shown as mean ± SD. *** P < 0.001 vs. Control.

Neurological function was evaluated 24 hours after reperfusion using the Bederson neurological deficit scale. The group receiving the control (FBS vehicle) exhibited the highest mean NDS, indicating severe neurological deficits. The miR-control EV group showed a reduction in NDS compared with the control group, but this difference did not reach statistical significance. The miR-146a-treated rats demonstrated a greater reduction in NDS than the control (P <0.0001) and the miR-control EV group (P = 0.0007) (Figure 3A). 

The results suggest that miR-control EVs did not significantly change outcomes compared with controls, but that miR-146a-enriched EVs induced a significant neuroprotective effect and improved neurological outcomes after MCAO.

### 4.5. Effects of miR-146a-Enriched Extracellular Vesicles on Regional CBF in MCAO

During ischemia and throughout reperfusion, regional CBF was continuously monitored. The data showed that MCAO resulted in significant reductions in CBF in all groups during occlusion. After reperfusion, the CBF in the FBS vehicle group decreased and remained decreased, with only partial recovery after 120 minutes.

Animals administered miR-control EVs tended to have improved CBF recovery at 30 and 60 minutes after reperfusion when compared with controls, but it is important to specify that these differences were not statistically significant, and by 120 minutes, CBF had not returned toward normal values.

Remarkably, the miR-146a-enriched EV treatment resulted in substantial recovery of cerebral blood flow. The rats that received miR-146a-enriched EVs had significantly higher CBF values at both 30 and 60 minutes post-reperfusion (P < 0.0001), and CBF levels recovered to near-baseline levels by the 120-minute time point (P < 0.0001) (Figure 3B). 

Overall, although EV treatment without enrichment did not significantly enhance recovery from reperfusion, treatment enriched with miR-146a significantly improved CBF with nearly complete restoration of the regional CBF following ischemic injury.

### 4.6. Effects of miR-146a-Enriched Extracellular Vesicles on Cytokine Production in Splenocytes

The levels of cytokines were measured in cultured splenocytes that were isolated 24 hours after MCAO. In splenocytes from the PBS control group, there was significantly elevated secretion of the pro-inflammatory cytokines TNF-α, IFN-γ, and IL-6. Treatment with miR-control EVs showed modest but statistically significant decreases in TNF-α, IFN-γ, and IL-6 levels and increases in IL-10 compared with PBS (P = 0.0469, P = 0.0447, P = 0.0452, 0.0473, respectively), which suggests that EVs alone have some influence in reducing inflammation; however, there were no significant observed differences for TGF-β between PBS and miR-control EVs (P = 0.0626).

**Figure 4.**
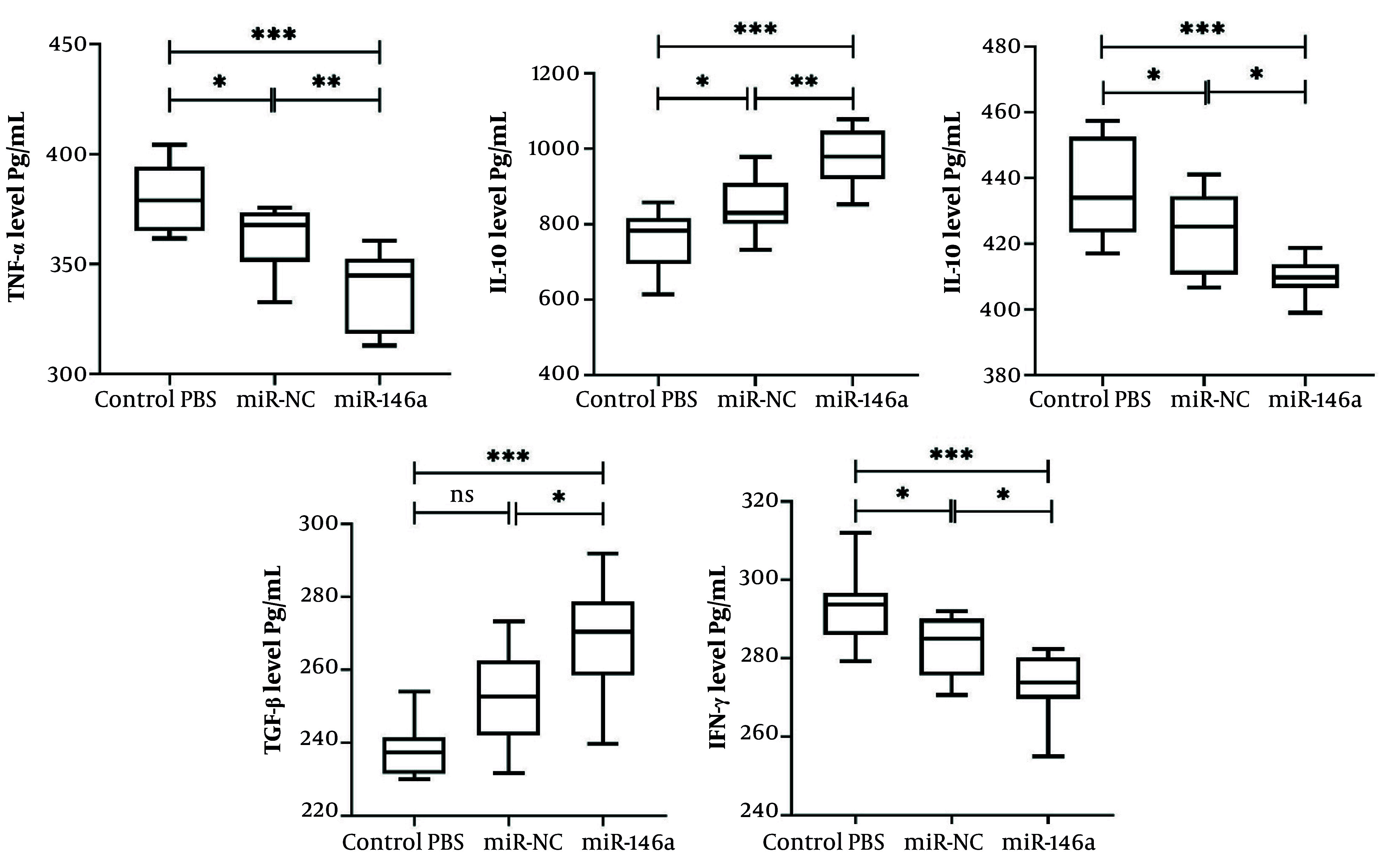
Impact of miR-146a on cytokine production in splenocytes. Levels of cytokines were assessed in the supernatant of cultured splenocytes, isolated at 24 hours post-MCAO, by ELISA. Treatment with miR-146a significantly decreased pro-inflammatory cytokines (TNF-α, IFN-γ, and IL-6) and elevated the production of anti-inflammatory cytokines (IL-10 and TGF-β) compared with the control and miR-NC groups. Data are presented as mean ± SD (n=10 each group). * P < 0.05, ** P < 0.01, *** P < 0.001. Abbreviations: ns, not significant; EV, extracellular vesicle; IL, interleukin; TGF, transforming growth factor-beta; TNF, tumor necrosis factor; IFN, interferon.

Conversely, miR-146a-enriched EV treatment had a more pronounced immunomodulatory effect, where splenocytes from this group had significantly decreased levels of TNF-α, IFN-γ, and IL-6 compared with control PBS (P < 0.0001) and miR-control EVs (P = 0.0067, P = 0.0319, P = 0.0303, respectively). Moreover, miR-146a-enriched EVs significantly increased levels of the anti-inflammatory cytokines TGF-β and IL-10 compared with both control PBS (P <0.0001) and miR-control EVs (P = 0.0228, P = 0.0014, respectively) (Figure 4). 

Overall, these results suggest that although the administration of EVs reduces levels of splenocyte-derived pro-inflammatory cytokines, the addition of miR-146a significantly enhances the anti-inflammatory profile through strong suppression of pro-inflammatory mediators and upregulation of immunoregulatory cytokines.

### 4.7. Effects of miR-146a-Enriched EVs on Cytokine Expression in the Brain

The levels of inflammatory and anti-inflammatory cytokines in the ischemic hemispheres were measured using qRT-PCR. When compared with the PBS control, expression of TNF-α, IFN-γ, and IL-6 transcripts was significantly decreased in ischemic brain tissue. miR-control EVs significantly decreased these pro-inflammatory cytokines compared with PBS (P = 0.001, P = 0.003, and P = 0.0009, respectively).

In addition, miR-146a-enriched EVs showed an even greater decrease. There were significantly greater decreases in TNF-α, IFN-γ, and IL-6 in rats that received miR-146a-enriched EVs as compared with PBS (P < 0.0001 for all) and in TNF-α and IL-6 as compared with miR-control EVs (P = 0.0008 and P = 0253, respectively).

**Figure 5.**
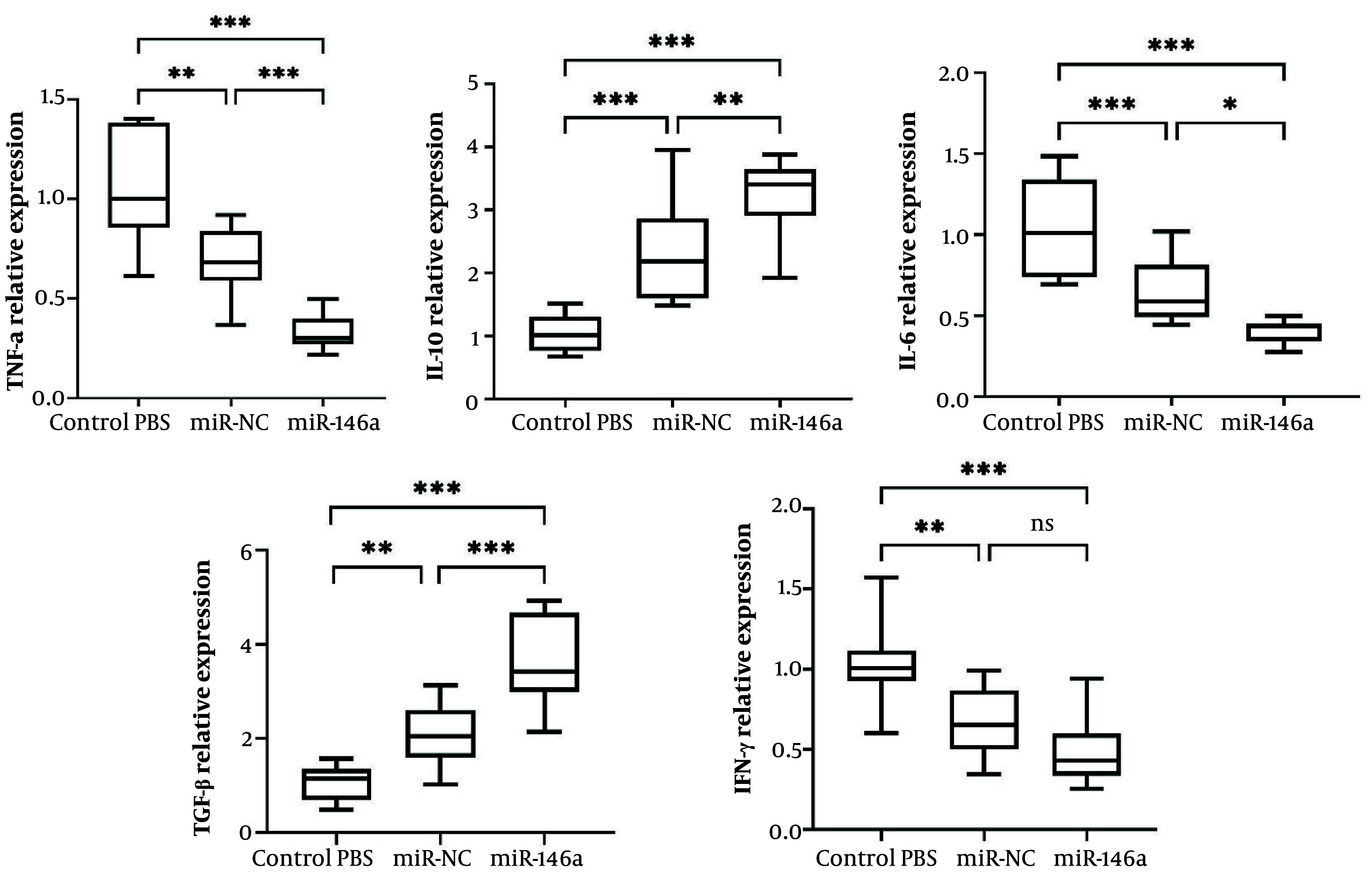
Effects of miR-146a on cytokine expression in the ischemic brain. miR-146a significantly reduced pro-inflammatory cytokines (TNF-α, IFN-γ, and IL-6) and increased anti-inflammatory cytokines (IL-10 and TGF-β), compared with the PBS and miR-control EV groups. Data are presented as mean ± SD (n = 10 per group). *** P < 0.001, ** P < 0.01, * P < 0.05. Abbreviations: EV, extracellular vesicle; IL, interleukin; TGF, transforming growth factor; TNF, tumor necrosis factor; SD, standard deviation.

Additionally, both TGF-β and IL-10 anti-inflammatory cytokines were significantly increased in the miR-control EV group compared with PBS (P = 0.008 and P < 0.0001). In addition, it is important to note that miR-146a further enhanced this response, as TGF-β and IL-10 expression were significantly higher than in the PBS group (P < 0.0001 for both) and than in the miR-control EV group (P = 0.0001 and P = 0.0035) (Figure 5). 

Overall, the data suggest that EV treatment alleviates the post-ischemia inflammatory response in the brain, and that miR-146a significantly enhances the effects of treatment by suppressing pro-inflammatory mediators and stimulating anti-inflammatory cytokines.

### 4.8. Effects of miR-146a-Enriched Extracellular Vesicles on the IRAK1/TRAF6/NF-κB Signaling Pathway in Ischemic Brain Tissue

**Figure 6.**
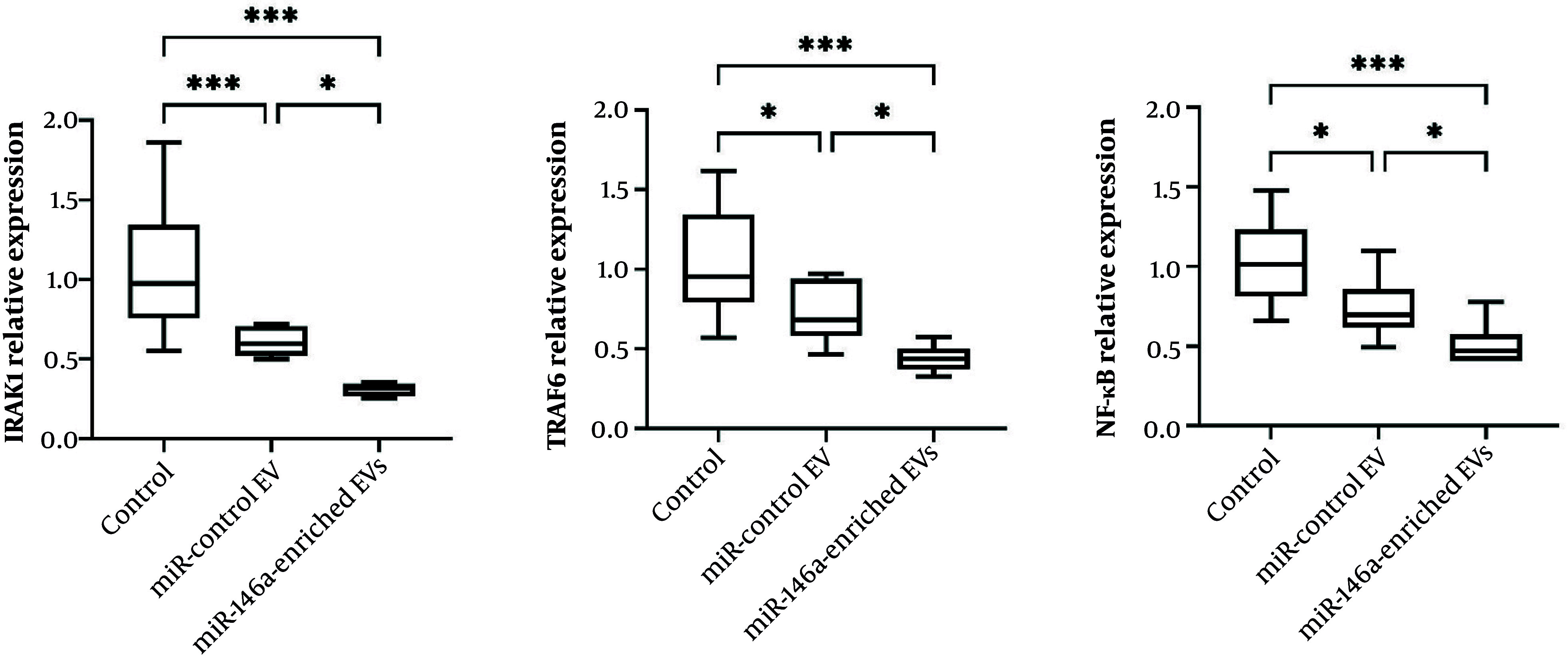
miR-146a-enriched EVs suppress IRAK1, TRAF6, and NF-κB signaling in ischemic brain tissue. Expression levels of core components in the IRAK1, TRAF6, and NF-κB signaling pathway in the ischemic brain at 24 h after reperfusion. The treatment with miR-146a-enriched EVs dramatically downregulated the expression of IRAK1, TRAF6, and NF-κB. Data are presented as mean ± SD (n = 10 per group). *** P < 0.001, ** P < 0.01, * P < 0.05. Abbreviations: EV, extracellular vesicle; IRAK1, interleukin-1 receptor-associated kinase 1; TRAF6, tumor necrosis factor receptor-associated factor 6; NF-κB, nuclear factor kappa B.

To explore the molecular mechanism of miR-146a-enriched EVs in suppressing neuroinflammation, we analyzed the expression of important mediators of the IRAK1, TRAF6, and NF-κB pathway in the ischemic brain. As illustrated in Figure 6, the rats administered miR-146a-enriched EVs showed a significant attenuation of IRAK1 and TRAF6 mRNA expression in comparison with the PBS-treated control group (P < 0.0001 for both) and the miR-control EV group (P = 0.0257 and P = 0.0241, respectively).

In addition, expression of NF-κB was dramatically inhibited after miR-146a-enriched EV treatment. The expression of NF-κB was markedly reduced in the miR-146a-EV group compared with the PBS-treated control group (P < 0.0001) and the miR-control EV group (P = 0.0464).

Altogether, our results indicate that miR-146a-enriched EVs potently target and suppress the IRAK1, TRAF6, and NF-κB inflammatory signaling pathway in the ischemic brain, which mechanistically substantiates their demonstrated anti-inflammatory and neuroprotective effects.

## 5. Discussion

Ischemic stroke continues to be a leading cause of death and long-lasting disability around the world. The standard of care (i.e., intravenous thrombolysis with alteplase and mechanical thrombectomy for large vessel occlusions) is time-sensitive and poses risks (1, 3). Therefore, there is an unmet clinical need for adjunctive therapies to prolong the therapeutic window and optimize neurological recovery while minimizing complications. The present study investigated the therapeutic capacity of BMSC-EVs enriched with miR-146a in a rat model of MCAO.

Data indicated that miR-146a-EVs significantly enhanced neurological recovery, reflected in reduced Bederson scores compared with both vehicle and miR-control EVs. Moreover, these vesicles showed more robust and complete recovery of CBF following reperfusion, and exhibited substantial anti-inflammatory properties, reflected by reduced pro-inflammatory cytokines (TNF-α, IFN-γ, IL-6) and enhanced anti-inflammatory mediators (IL-10, TGF-β). Overall, these findings suggest that miR-146a-EVs may provide a novel therapeutic option for attenuating neuroinflammation and improving functional recovery following ischemic stroke.

Neuroinflammation represents a double-edged sword in stroke pathology (4). Activated microglia, immune cell infiltration, and maladaptive cytokine release drive secondary injury, accounting for neuronal death and further clinical deficits (20). This understanding has led researchers to target modulation of the inflammatory context rather than complete inhibition of the immune response.

The present study provides direct mechanistic evidence that the anti-inflammatory effects of miR-146a-enriched EVs are mediated, at least in part, through suppression of the IRAK1, TRAF6, and NF-κB signaling pathway. miR-146a is a known negative regulator of innate immune signaling that directly targets IRAK1 and TRAF6, two key adaptor molecules involved in NF-κB activation downstream of Toll-like receptor and interleukin-1 receptor signaling (21). In the present study, miR-146a-enriched EV treatment notably suppressed the expression of IRAK1, TRAF6, and NF-κB in the ischemic brain, attenuating the transcriptional activation of pro-inflammatory cytokines. These mechanistic changes were mirrored by a decrease in TNF-α, IFN-γ, and IL-6 and an increase in anti-inflammatory compounds, including IL-10 and TGF-β. Inhibition of NF-κB signaling by miR-146a-enriched EVs might contribute to the interruption of this vicious self-propagating inflammatory cascade that causes secondary neuronal damage after ischemic stroke. These pathway-level validations further support the biological feasibility of miR-146a-EVs as a precise therapeutic intervention for post-stroke neuroinflammation.

A great deal of interest in regenerative neurology has centered on cell-based therapies, specifically the use of MSCs. Whole-cell transplantation has its challenges, however, including the risk of uncontrolled differentiation, immunogenicity, and tumorigenesis (5). Extracellular vesicles mitigate many of the adverse effects of whole-cell transplantation, acting naturally as protein, lipid, and nucleic acid carriers that replicate the benefits of their parent cells (22).

The present results demonstrated that EVs alone (miR-control EVs) resulted in modest improvements, including partial inhibition of pro-inflammatory cytokines. These outcomes were again significantly improved with enrichment of EVs using miR-146a, suggesting a critical engineering strategy to enhance the efficacy of EVs based on their cargo. Indeed, the benefits generated by miR-146a-enriched EVs have been documented in animal studies of spinal cord injury (13), retinal degeneration (12), and acute lung injury (14), supporting a broader translatable methodology across different inflammatory conditions.

A particularly important finding from this study was that miR-146a-EVs were able to significantly restore CBF following reperfusion, almost to baseline levels within 120 minutes. Restoring perfusion is key to neuronal survival because a lack of it can lead to larger infarct size and poorer outcomes (23). The mechanisms of CBF improvement by miR-146a-EVs have not been fully explored; however, their anti-inflammatory effects may have stabilized the neurovascular unit, improved endothelial dysfunction, and decreased microvascular obstruction. Previous work has shown that inflammatory cytokines lead to blood-brain barrier (BBB) disruption and increased impairment of cerebrovascular reactivity (4, 24). Thus, miR-146a-EVs may promote vascular integrity and allow better reperfusion by blocking cytokines. The fact that miR-146a-enriched EVs improve cerebral blood flow is a particularly novel and translatable result of this study. Although many interventions in experimental stroke focus predominantly on neuronal survival or on limiting infarct size, impaired reperfusion at the microvascular level (commonly known as the no-reflow phenomenon) is an important contributor to secondary damage and poor neurological outcome (23). miR-146a is known to suppress NF-κB-dependent endothelial activation, which may reduce leukocyte adhesion, cytokine-mediated vasoconstriction, and microvascular plugging, thereby facilitating more effective reperfusion (10).

The clinical application of miR-146a-EV therapy holds great potential. In contrast to thrombolytics or mechanical thrombectomy, both of which require a narrow therapeutic window and specialized training, EV therapy could potentially be delivered later in the therapeutic range. Furthermore, intravenous delivery, used in this investigation, is a minimally invasive route for delivery that can be easily adapted into the clinical process. However, there are challenges. There remains a need to determine how to standardize EV isolation, determine dosing, and standardize quality control for reproducibility and safety. Furthermore, before clinical trials, the potential for any off-target immune modulation will require testing in larger animal models.

Although the findings are remarkable, there are a few limitations that must be discussed. First, the present study evaluated outcomes at a single acute time point (24 hours post-reperfusion), which limits conclusions regarding the durability of neurological recovery, sustained cerebral blood flow improvement, and long-term neurovascular remodeling. Whether the observed CBF restoration persists beyond the acute phase or translates into reduced infarct volume and chronic functional improvement remains to be determined. Second, the study used male rats exclusively, and it is known that the response to immune manipulation can differ by sex and may influence treatment efficacy. Third, while cytokine modulation was clearly observed in the study, the specific molecular targets and downstream activation of miR-146a-EVs in the ischemic brain need to be further explored.

Future studies may include longer timeframes to explore chronic recovery, both males and females as a group, and advanced imaging and histological measures to quantify infarct volume, neurogenesis, and synaptic remodeling. Further, combinatorial approaches with miR-146a-EVs, in conjunction with currently available reperfusion therapies, may produce greater synergistic benefit.

### 5.1. Conclusions

In conclusion, the current study offers strong preclinical evidence that miR-146a-enriched MSC-EVs are capable of reducing neuroinflammation, improving cerebral reperfusion, and enhancing neurological outcomes following ischemic stroke in the rat model. By utilizing the anti-inflammatory and reparative effects of miR-146a, this approach addresses a significant void in stroke therapy today. While additional studies are needed to assess the sustained efficacy and safety of miR-146a-EVs, they represent a potential for clinical translation in stroke therapy.

## Data Availability

The data that support the findings of this study are available from the corresponding author upon reasonable request.
